# A pan-cancer analysis of the prognostic and immunological role of β-actin (ACTB) in human cancers

**DOI:** 10.1080/21655979.2021.1973220

**Published:** 2021-09-04

**Authors:** Yuxi Gu, Shouyi Tang, Zhen Wang, Luyao Cai, Haosen Lian, Yingqiang Shen, Yu Zhou

**Affiliations:** State Key Laboratory of Oral Diseases, National Clinical Research Center for Oral Diseases, Chinese Academy of Medical Sciences Research Unit of Oral Carcinogenesis and Management, West China Hospital of Stomatology, Sichuan University, Chengdu, China

**Keywords:** ACTB, biomarker, pan-cancer, prognosis, immune analysis, functional relevance

## Abstract

Beta-actin (ACTB), a highly conserved cytoskeleton structural protein, has been regarded as a common housekeep gene and used as a reference gene for years. However, accumulating evidence indicates that ACTB is abnormally expressed in multiple cancers and hence changes the cytoskeleton to affect the invasiveness and metastasis of tumors. This study aimed to investigate the function and clinical significance of ACTB in pan-cancer. The role of ACTB for prognosis and immune regulation across 33 tumors was explored based on the datasets of gene expression omnibus and the cancer genome atlas. Differential expression of ACTB was found between cancer and adjacent normal tissues, and significant associations was found between ACTB expression and prognosis of tumor patients. In most cancers, ACTB expression was associated with immune cells infiltration, immune checkpoints and other immune modulators. Relevance between ACTB and metastasis and invasion was identified in various types of cancers by CancerSEA. Moreover, focal adhesion and actin regulation-associated pathways were included in the functional mechanisms of ACTB. The expression of ACTB was verified by quantitative real-time polymerase chain reaction. Knockdown of ACTB inhibited head and neck squamous carcinoma cell migration and invasion by NF-κB and Wnt/β-catenin pathways. Our first pan-cancer study of ACTB offers insight into the prognostic and immunological roles of ACTB across different tumors, indicating ACTB may be a potential biomarker for poor prognosis and immune infiltration in cancers, and the role of ACTB as a reference gene in cancers was challenged.

## 1. Introduction

Cancer has been known as the top killer threatening human health for decades all over the world [1]. In spite of considerable effort , the five-year survival rate is still unsatisfying [1,2]. Considering the complexity of tumorigenesis and currently successful application of cancer biomarkers [3,4], it is important to be curious about any gene of interest and explore its prognostic value and underlying molecular mechanisms in cancers.

Beta-actin (ACTB), a highly conserved cytoskeleton structural protein associated with cell growth and cell migration [5], has been considered as an endogenous housekeeping gene and a reference gene in cells and tissues for years [6–8]. However, emerging evidence suggests that ACTB is differentially expressed and plays a crucial role in multiple human diseases, especially cancers [9–14]. The cytoskeleton structure and the actin microfilaments system are known to regulate tumor cell adhesion and locomotion, which are important in tumor growth and metastasis [15]. ACTB polymerization, localization, cytoskeleton formation and overexpression might promote colon adenocarcinoma cell motility and metastasis [16,17]. Different distribution of ACTB at the perinucleus region and cell leading edge could influence the polarity and plasticity of tumor cell locomotion, regulating the tumor metastasis [18]. Additionally, upregulation and aggregation of ACTB in the pseudopodia might facilitate tumor cell invasion [19]. ACTB protein levels were significantly altered in renal cell carcinoma cell lines and tissues compared to normal control [20]. ACTB was deregulated in different tumor invasiveness degrees and TNM stages of hepatocellular carcinoma and 3’-UTR of ACTB has been demonstrated to play an important role in the process of HCC development [21–23]. However, despite the great resource of big clinical data, the relationship of ACTB and pan-cancer remains unknown.

Our study carried out a pan-cancer analysis of ACTB based on The Cancer Genome Atlas (TCGA) and Gene Expression Omnibus (GEO) databases for the first time to explore the common role of ACTB in pan-cancer. We investigated ACTB expression in normal tissues and pan-cancer. We also explored prognostic value, genetic alteration, immune correlation, relevant cellular pathway and function of ACTB to reveal the potential molecular pathogenesis of multiple cancers, challenging the role of ACTB as a reference gene for cancers and presenting a potential prognosis biomarker and immunotherapy target.

## 2. Materials and methods

### 2.1. ACTB expression analysis

Human Protein Atlas (HPA) (https://www.proteinatlas.org/) is a database of proteins in human organs, tissues and cells based on multiple omics approaches [24,25]. ACTB mRNA expression in normal tissues was explored using HPA database. Furthermore, the immunohistochemistry images of ACTB proteins were acquired from the atlas modules of tissue and pathology.

The protein expression of ACTB in normal and cancer tissues were retrieved from genecards database (https://www.genecards.org/) [26].

Oncomine (www.oncomine.org) is a cancer microarray database for gene expression analysis based on databases and published papers [27]. ACTB expression was investigated on Oncomine, and cutoff criteria was set as P < 0.05 and fold change >2.

ACTB expression data in cancer and adjacent normal tissues in TCGA database was downloaded from UCSC Xena (https://xenabrowser.net/) [28]. The abbreviations and full names of the 33 cancers in TCGA are presented in Table 1. ACTB expression in different pathological stages (stage I ~ IV) of different cancers was obtained using Gene Expression Profiling Interactive Analysis 2 (GEPIA2) [29].

**Table 1.** Abbreviations and full names of all TCGA cancers

Abbreviation	Full name
ACC	Adrenocortical carcinoma
BLCA	Bladder urothelial carcinoma
BRCA	Breast invasive carcinoma
CESC	Cervical squamous cell carcinoma
CHOL	Cholangiocarcinoma
COAD	Colon adenocarcinoma
DLBC	Diffuse large B cell lymphoma
ESCA	Esophageal carcinoma
GBM	Glioblastoma multiforme
HNSC	Head and neck squamous cell carcinoma
KICH	Kidney chromophobe
KIRC	Kidney renal clear cell carcinoma
KIRP	Kidney renal papillary cell carcinoma
LAML	Acute myeloid leukemia
LGG	Brain lower grade glioma
LIHC	Liver hepatocellular carcinoma
LUAD	Lung adenocarcinoma
LUSC	Lung squamous cell carcinoma
MESO	Mesothelioma
OV	Ovarian serous cystadenocarcinoma
PAAD	Pancreatic adenocarcinoma
PCPG	Pheochromocytoma and paraganglioma
PRAD	Prostate adenocarcinoma
READ	Rectum adenocarcinoma
SARC	Sarcoma
SKCM	Skin cutaneous melanoma
STAD	Stomach adenocarcinoma
TGCT	Testicular germ cell tumors
THCA	Thyroid carcinoma
THYM	Thymoma
UCEC	Uterine corpus endometrial carcinoma
UCS	Uterine carcinosarcoma
UVM	Uveal melanoma

### 2.2. Survival prognosis analysis

The survival relevance of ACTB across all TCGA tumors were gained from GEPIA2 [29]. We used cutoff value 50% to split the high-ACTB-expression and low-ACTB-expression cohorts. We also obtained the survival plots utilizing GEPIA2.

Kaplan–Meier Plotter (http://kmplot.com/analysis/) is a database for assessing the prognosis values of human genes in more than 10,000 cancer samples. The correlation between ACTB and the survival of different cancers in pan-cancer were also analyzed with the Kaplan–Meier Plotter [30].

Prognostic value of ACTB in GEO datasets (GSE17536 [31–34], GSE1456-GPL96 [35,36], GSE13507 [37,38], GSE19615 [39], GSE30929 [40], GSE4412-GPL96 [41], GSE9893 [42], GSE14764 [43], GSE16560 [44], GSE9195 [45,46] and GSE31210 [47,48]) were determined using Prognoscan database [49].

### 2.3. Genetic alteration analysis

The cBioPortal (http://www.cbioportal.org), a comprehensive database of cancer genomics datasets [50], was used to analyze genetic alteration of ACTB. We explored the copy number alteration (CNA) and mutation status of ACTB across all TCGA tumors using cBioPortal. The results of the alteration frequency, mutation type and CNA in multiple cancers were obtained from the ‘Cancer Types Summary’ module.

### 2.4. Immune infiltration analysis

The correlation between ACTB expression and immune infiltration in pan-cancer was investigated using Tumor IMmune Estimation Resource 2 (TIMER2) (http://timer.cistrome.org/) [51]. Cancer associated fibroblasts, macrophages, endothelial cells, monocytes and CD8 + T-cells were selected. The results were visualized as heatmaps and scatter plots.

The immune infiltration data for stage-specific immune infiltration analysis was obtained from EPIC server (http://epic.gfellerlab.org/) [52].

### 2.5. Correlation analysis

TIMER2 was used to estimate the associations between ACTB and immune checkpoints, mismatch repair (MMR) signatures. Tumor-Immune System Interaction DataBase (TISIDB) [53] (http://cis.hku.hk/TISIDB/) was utilized to explore the relationships of ACTB and immune modulators in pan-cancer. The ‘pheatmap’ package was used to demonstrate the results of the stage-specific immune checkpoint correlation.

### 2.6. Functional relevance analysis

Single-cell RNA sequencing (ScRNA-seq) offered an unparalleled condition for us to investigate the functional specificity of cancer cells. CancerSEA (http://biocc.hrbmu.edu.cn/CancerSEA/) is a database for functional states of cancer cells at single-cell level [54]. The functional state of ACTB in multiple cancers was explored using CancerSEA. Correlations between ACTB and functional state were filtered by a correlation strength >0.3 and P value <0.05.

### 2.7. ACTB-related gene enrichment analysis

The STRING [55] (https://string-db.org/) was used to investigate the protein interaction network of ACTB. Main parameters were set as follows: meaning of network edges as ‘evidence’, minimum required interaction score as ‘Low confidence (0.150)’, active interaction sources as ‘experiments’ and max number of interactors to show as ‘no more than 50 interactors’. As a result, experimentally determined ACTB-interacted proteins and relevant interaction network were gained.

GEPIA2 was applied to get the top 100 ACTB-correlated genes based on TCGA data. Pearson correlation coefficient analysis between ACTB and the top 5 correlated genes was performed using the ‘correlation analysis’ module of GEPIA2. The log2 TPM, correlation coefficient (R) and P value were demonstrated. Additionally, TIMER2 was applied to estimate the correlations between ACTB and the top five genes. We then used draw venn diagram (http://bioinformatics.psb.ugent.be/Webtools/Venn/) to perform intersection analysis of the ACTB-interacted and correlated genes.

Kyoto encyclopedia of genes and genomes (KEGG) pathway analysis was performed on DAVID [56]. The results were visualized applying the ‘tidyr’ and ‘ggplot2’ R packages. Moreover, the “clusterProfiler” R package was used to perform Gene ontology (GO) enrichment analysis. The results of Biological process (BP), Molecular function (MF) and Cellular component (CC) were visualized through the cnetplot (circular = F, colorEdge = T, node_label = T). P < 0.05 was regarded as statistically significant. The R language software version used in this study was [R-4.0.3, 64-bit] (https://www.r-project.org/).

### 2.8. *Verifying the expression and function of ACTB* in vitro

Normal head and neck cell lines HOK and NOK and HNSC cell lines SCC9, SCC25, CAL33 and H400 were used. The SCC9 and SCC25 cell lines were gained from American-type culture collection (ATCC; Manassas, VA, USA). The CAL33 cell line was obtained from the NIDCR Oral and Pharyngeal Cancer Branch cell collection. The H400 cell line was established at Bristol Dental School, University of Bristol, UK [57]. All of the cell lines used in this study were stored in state key laboratory of oral diseases (West China Hospital of Stomatology, Sichuan University). Firstly, the total RNA was isolated form HOK, NOK, SCC9, SCC25, CAL33 and H400 cells using TR250 total RNA extraction kit (Tianmobio, Beijing, China) and reverse transcribed into cDNA through Prime-Script RT reagent Kit with gDNA Eraser (Takara, Japan) according to manufacturer’s instructions. Subsequently, the expression of ACTB was detected by Real-Time Quantitative Polymerase Chain Reaction (RT-qPCR). We used 2− ΔΔ CT method to quantify the relative gene expression and GAPDH was applied as the internal control. Below the list of primers, GAPDH: forward primer 5′-CCTGTTCGACAGTCAGCCG-3′ and reverse primer 5′-CGACCAAATCCGTTGACTCC-3′, ACTB: forward primer 5′- AGCGAGCATCCCCCAAAGTT-3′ and reverse primer 5′- GGGCACGAAGGCTCATCATT-3′, CTNNB1: forward primer 5′- AAAGCGGCTGTTAGTCACTGG-3′ and reverse primer 5′- CGAGTCATTGCATACTGTCCAT-3′, RHOA: forward primer 5′- AGCCTGTGGAAAGACATGCTT-3′ and reverse primer 5′- TCAAACACTGTGGGCACATAC-3′, NFKB1: forward primer 5′- AACAGAGAGGATTTCGTTTCCG-3′ and reverse primer 5′- TTTGACCTGAGGGTAAGACTTCT-3′, RELA: forward primer 5′- ATGTGGAGATCATTGAGCAGC-3′ and reverse primer 5′- CCTGGTCCTGTGTAGCCATT-3′. GraphPad Prism 7 software was used and unpaired Student’s t-tests was conducted. P < 0.05 was considered statistically significant.

The sequences of human ACTB-targeting small interfering RNA (SiRNA) (RiboBio, Guangzhou, China) are CCAGCCATGTACGTTGCTA for siRNA1, GGGACGACATGGAGAAAAT for siRNA2 and TGCGTGACATTAAGGAGAA for siRNA3. Negative Control siRNA (RiboBio) was used as the nontargeted siRNA control. SCC25 and CAL33 cells were seeded into 6-well culture plates and transfected using riboFECT CP Transfection Kit(166 T) (RiboBio, Guangzhou, China) following the manufacturer’s protocol. The efficiency of ACTB knockdown was assessed by RT-qPCR.

Transwell assay were performed in 8-mm Transwell (#353,097, FALCON, USA). For the migration assay, we added 200 μL of HNSC cell suspension (5 × 10^5^ /mL) in the upper chamber, and 500 μL of complete medium in the lower chamber. The cells were incubated at 37°C for 24 h. The membranes were fixed in 4% paraformaldehyde and stained using crystal violet solution. For the invasion assay, the transwell were coated with Matrigel (BD Biosciences, NY, USA) and cells were incubated at 37°C for 24 h.

## 3. Results

The role of ACTB in pan-cancer including expression profile, overall survival analysis, immune landscape and functional relevance was explored. ACTB expression was increased in various cancers and high expression of ACTB was correlated with poor patient prognosis in different cancers. ACTB was correlated with immune infiltration and immune checkpoints. We also found knockdown of ACTB in HNSC cell lines would inhibit tumor cell migration and invasion.

### 3.1. ACTB expression analysis in human normal tissues

**Figure 1.**
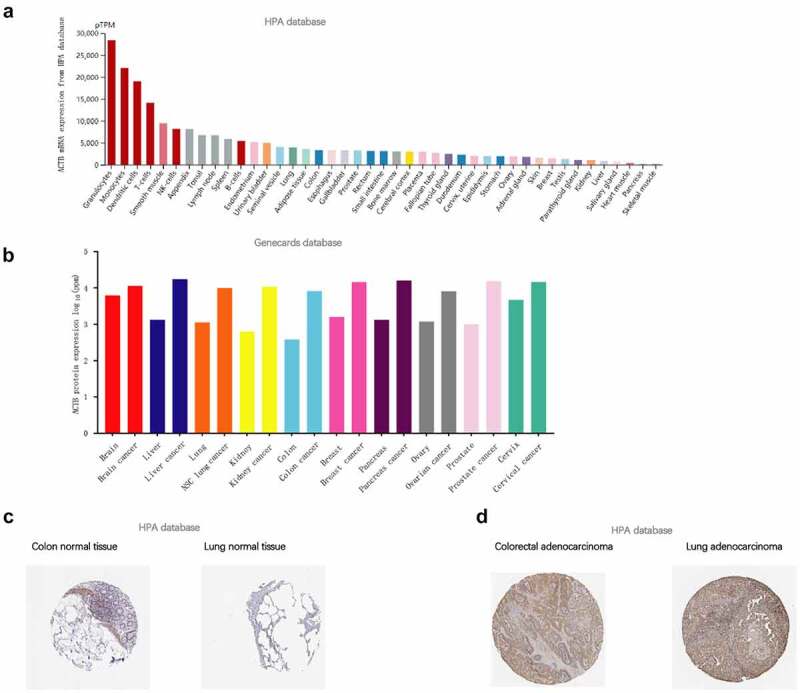
**ACTB expression condition in normal and tumor tissues**. (a) ACTB mRNA expression condition in normal tissues based on human protein atlas (HPA) database. (b) ACTB protein expression condition in normal and cancer tissues from the genecards database. (c) ACTB expression in normal colon and lung tissues from HPA database. (d) ACTB expression in colorectal and lung cancer tissues from HPA database

To illustrate ACTB expression condition in normal tissues, we investigated ACTB expression in different normal tissues using HPA database. Results showed that ACTB mRNA expression was enriched in blood, lymphoid, gastrointestinal tract and muscle tissues (Figure 1(a)). ACTB protein was differentially expressed in normal and cancer tissues, at higher levels in brain, liver, lung, kidney, colon, breast, pancreas, ovarian, prostate and cervical cancers (Figure 1(b)). ACTB protein was mainly located in cytoplasm and membrane and expressed lowly in in normal colon and lung tissues (Figure 1(c)). High ACTB expression was observed in corresponding cancer tissues (Figure 1(d)). The results of IHC were demonstrated in Table 2.

**Table 2.** Clinical information and relative immunohistochemistry results

Protein	Tissue	Histological type	Age		Gender	Location	Quantity	Intensity
ACTB	Colon	Normal tissue glandular cells		84	Female	Cytoplasmic/membranous	75%-25%	Moderate
ACTB	Colorectal cancer	Adenocarcinoma		84	Female	Cytoplasmic/membranous	>75%	Moderate
ACTB	Lung	Normal tissue		65	Male	Cytoplasmic/membranous	<25%	Moderate
ACTB	Lung cancer	Adenocarcinoma		65	Male	Cytoplasmic/membranous	>75%	Moderate

### 3.2. ACTB expression analysis in human cancers

**Figure 2.**
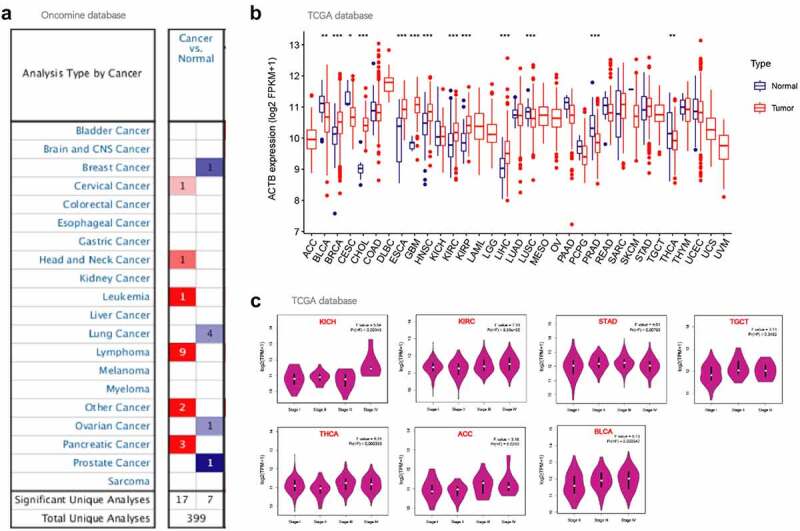
**ACTB expression in pan-cancer and different pathological stages**. (a) ACTB expression in various cancers in comparison with normal tissues using Oncomine. (b)The expression status of the ACTB in multiple cancers using tumor immune estimation resource 2 (TIMER2). * P < 0.05; ** P < 0.01; *** P < 0.001. (c) ACTB expression in different pathological stages (stage I~ IV) of kidney Chromophobe (KICH), kidney renal clear cell carcinoma (KIRC), stomach adenocarcinoma (STAD), testicular germ cell tumors (TGCT), thyroid carcinoma (THCA), adrenocortical carcinoma (ACC) and bladder Urothelial Carcinoma (BLCA) using GEPIA2 based on the the cancer genome atlas (TCGA) data

We compared ACTB mRNA expression differences in tumor and normal tissues utilizing Oncomine database. ACTB expression was observed higher in cervical cancer, head and neck cancer, leukemia, lymphoma and pancreatic cancer compared with normal tissues (Figure 2(a)), and lower in breast cancer, lung cancer, ovarian cancer and prostate cancer. Then, the expression of ACTB mRNA across all TCGA cancers was investigated on TIMER2. ACTB expression in the tumor tissues of BRCA, CHOL, ESCA, GBM, HNSC, KIRC, KIRP, LIHC (P < 0.001) is higher compared to normal tissues (Figure 2(b)). While the expression of ACTB in cancer tissues of LUSC, PRAD (P < 0.001), BLCA and THCA (P < 0.01) is lower than the normal tissues.

We also applied GEPIA2 to explore the correlation of ACTB expression and the pathological stages of cancers. Significant correlation was observed in KICH, KIRC, STAD, TGCT, THCA, ACC and BLCA (Figure 2(c), all P < 0.05).

### 3.3. Survival analysis

**Figure 3.**
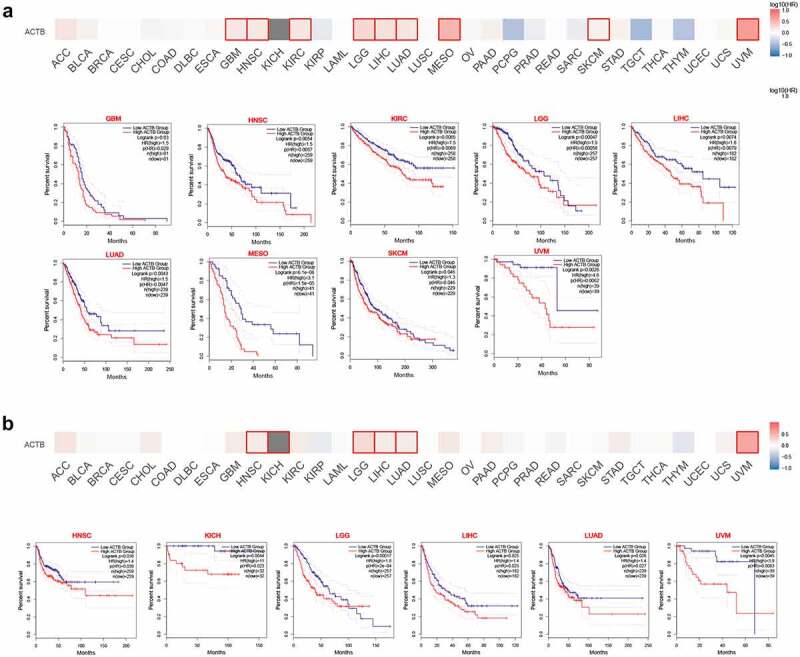
**Relationship between ACTB expression and patient prognosis of different cancers based on the TCGA data**. (a) OS map and Kaplan-Meier curves of TCGA cancers by ACTB gene expression using gene expression profiling interactive analysis 2 (GEPIA2). (b) DFS map and Kaplan-Meier curves of TCGA cancers by ACTB gene expression using GEPIA2

The cancer patients were divided into high-ACTB-expression and low-ACTB-expression groups and the association of ACTB expression with the patient prognosis in multiple cancers was explored based on TCGA and GEO datasets. As a result, We found high ACTB expression was correlated with poor overall survival (OS) for GBM (P = 0.03), HNSC (P = 0.0054), KIRC (P = 0.0065), LGG (P = 0.00047), LIHC (P = 0.0074), LUAD (P = 0.0043), MESO (P = 6.1e-06), SKCM (P = 0.046) and UVM (P = 0.0026) from TCGA datasets (Figure 3(a)). We also found (Figure 3(b)) high ACTB expression was associated with poor Disease-free survival (DFS) for HNSC (P = 0.038), KICH (P = 0.0044), LGG (P = 0.00017), LIHC (P = 0.025), LUAD (P = 0.026) and UVM (P = 0.0045).

Survival analysis by Kaplan-Meier plotter also showed a correlation of high ACTB expression and poor OS in BLCA (P = 0.013), HNSC (P = 0.0015), KIRC (P = 8.9e−06), LIHC (P = 0.0088), LUAD (P = 0.0023), LUSC (P = 0.029), READ (P = 0.032) and STAD (P = 0.038), while high expression of ACTB was found correlated with better OS in PCPG (P = 0.024), SARC (P = 0.022), THYM (P = 0.0011) and UCEC (P = 0.029) (Fig. S1A). Relapse-free survival (RFS) analysis (Fig. S1B) presented a correlation between high ACTB expression and poor prognosis for breast cancer (P = 0.024), LUAD (P = 0.043), ovarian cancer (P = 0.037), STAD (P = 0.038) and THCA (P = 0.01), while correlation between high ACTB and better RFS in BLCA (P = 0.027), esophageal squamous cell carcinoma (P = 0.026), KIRC (P = 0.023), PCPG (P = 0.024) and UCEC (P = 0.0075).

Based on GEO datasets using prognoscan, we found high ACTB expression was correlated with poor OS (Fig. S2A), RFS (Fig. S2B), disease-specific survival (DSS) (Fig. S2C), distant metastasis free survival (DMFS) (Fig. S2D), distant recurrence free survival (DRFS) (Fig. S2E) and DFS (Fig. S2F) in various cancers (All P < 0.05).

To clarify the relationship between ACTB expression and prognosis in different stages, stage-specific OS analysis was performed in the seven cancers with stage dependency of ACTB expression (Fig. S3). Most of the results were in line with former OS analysis. For ACC, BLCA, STAD, TGCT and THCA, no significant relationship was observed between ACTB expression and patient prognosis in low or high stage. For KIRC, high ACTB expression was related to poor prognosis in both low and high stage. However, for KICH, high ACTB expression was associated with poor prognosis in high stage while no significance was found in low stage.

### 3.4. Genetic alteration analysis

**Figure 4.**
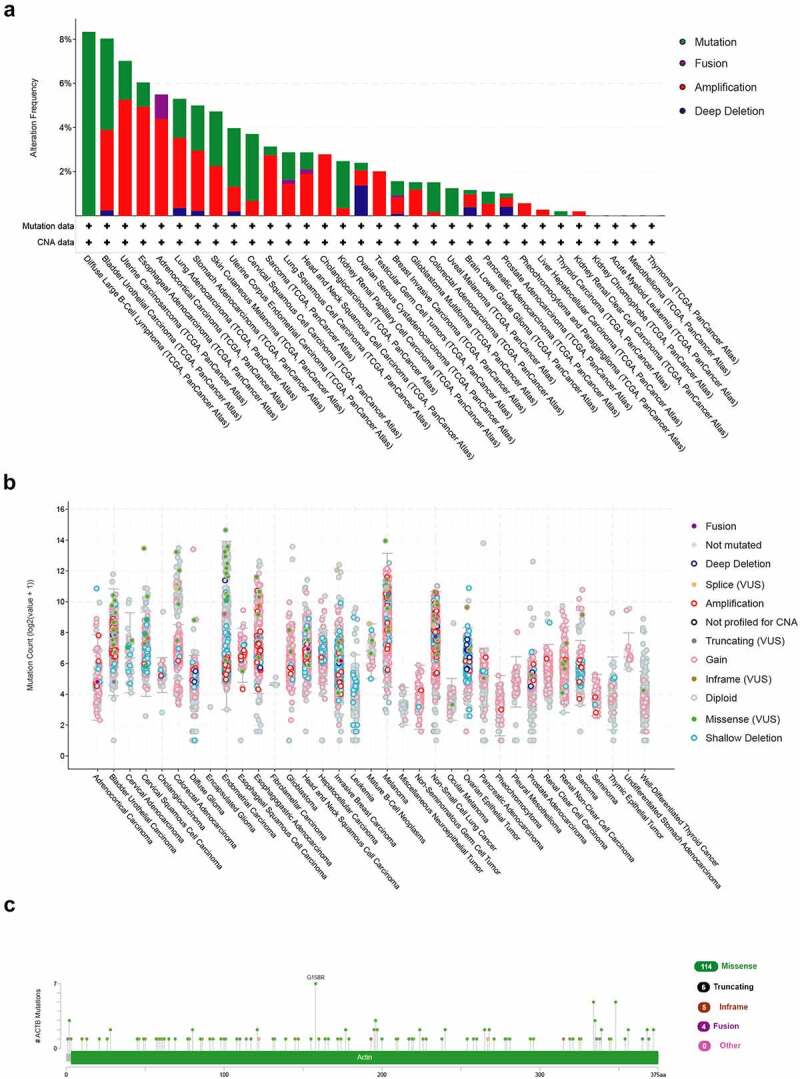
**Mutation feature of ACTB in pan-cancer from TCGA**. (a)The alteration frequency of ACTB in pan-cancer datasets from TCGA through the cBioPortal database. (b) Mutation count of ACTB across all TCGA cancers using cBioPortal. (c) Mutation site of ACTB in pan-cancer from cBioPortal

The genetic alteration status of ACTB in pan-cancer was explored using Cbioportal based on TCGA datasets. The highest alteration frequency of ACTB was showed in diffuse large b-cell lymphoma and bladder urothelial carcinoma, mostly mutation. Amplification was the dominating type in the uterine carcinosarcoma, esophageal adenocarcinoma and adrenocortical carcinoma (Figure 4(a)). The mutation count of ACTB in pan-cancer was shown in Figure 4(b). The types and sites of ACTB genetic alteration were demonstrated in Figure 4(c). From amino acids 0 to 585, 129 mutation sites were detected. The mutation sites contained 114 missense, 16 truncating, 5 inframe and 4 fusion mutations, and G158R was the most frequent one (Figure 4(c)).

### 3.5. Immune infiltration analysis

**Figure 5.**
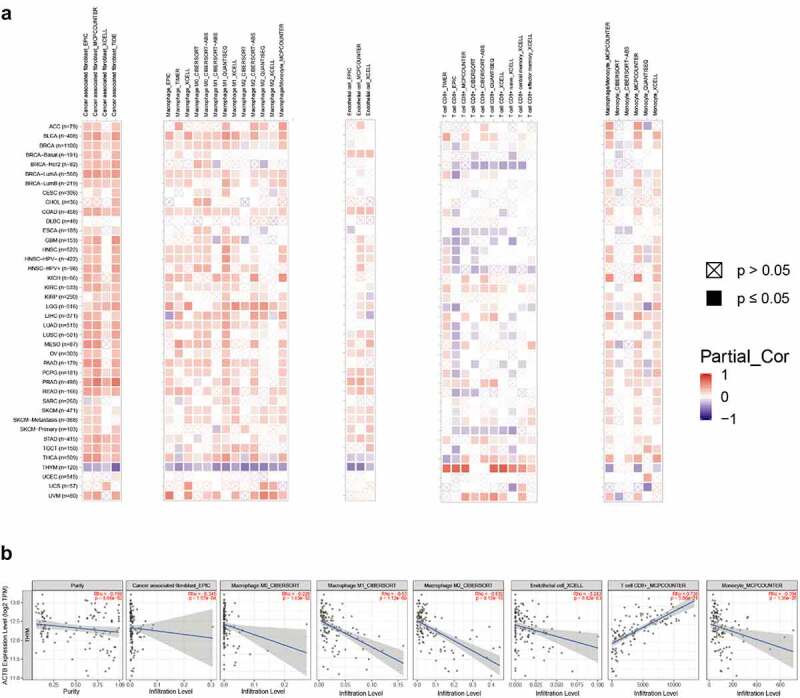
**Correlation analysis of ACTB and immune infiltration across all TCGA cancers using TIMER2**. (a)The correlations of ACTB expression and immune infiltration across all TCGA cancers by different algorithms through TIMER2. (b) The correlation of ACTB expression and immune infiltration in Thymoma (THYM) on TIMER2

Tumor-infiltrating immune cells play crucial roles in the initiation, progression and metastasis of cancers [58]. Herein, we used the TIMER2 to explore the relationship of ACTB expression and the infiltration level of various immune cells in all TCGA cancers. As a result, ACTB was positively correlated with infiltration levels of cancer associated fibroblasts (CAF), macrophages, endothelial cells and monocytes (Figure 5(a)). ACTB expression was also negatively correlated with immune infiltration of CD8 + T-cells in BRCA-Her2, ESCA, GBM, HNSC and SKCM−Primary, while positively correlated in KIRC and THYM. Intriguingly, we observed a quite significant correlation between ACTB expression and immune infiltration in THYM. The scatterplot data of THYM were presented in Figure 5(b). The ACTB expression level in THYM was negatively correlated with the infiltration level of CAF (Rho = −0.345, P = 1.57e-04, based on EPIC), macrophages M0(Rho = −0.228, P = 1.43e-02, based on CIBERSORT), macrophages M1(Rho = −0.53, P = −1.12e-09, based on CIBERSORT), macrophages M2(Rho = −0.532, P = 9.12e-10, based on CIBERSORT), endothelial cells(Rho = −0.243, P = 8.82e-03, based on XCELL) and monocyte(Rho = −0.394, P = 1.36e-05, based on MCPCOUNTER), while positively correlated with the infiltration level of CD8 + T cell (Rho = 0.738, P = 5.00e-21) based on the MCPCOUNTER algorithm.

In aforementioned 7 cancers with stage dependency in ACTB expression, stage- specific immune infiltration analysis was conducted. Stage-dependent correlation of ACTB expression and immune infiltration was found for B cells, CD8 + T cells, endothelial and NK cells in ACC, endothelial BLCA, CAFs in KICH, CD4 + T cells in KIRC, B cells, CD4+ cells and macrophages in STAD, B cells, CD4 + T cells, endothelial and macrophages in TGCT, and CD8+ and NK cells in THCA (Fig. S4).

### 3.6. Relationship between ACTB and immune checkpoints, immune modulators

**Figure 6.**
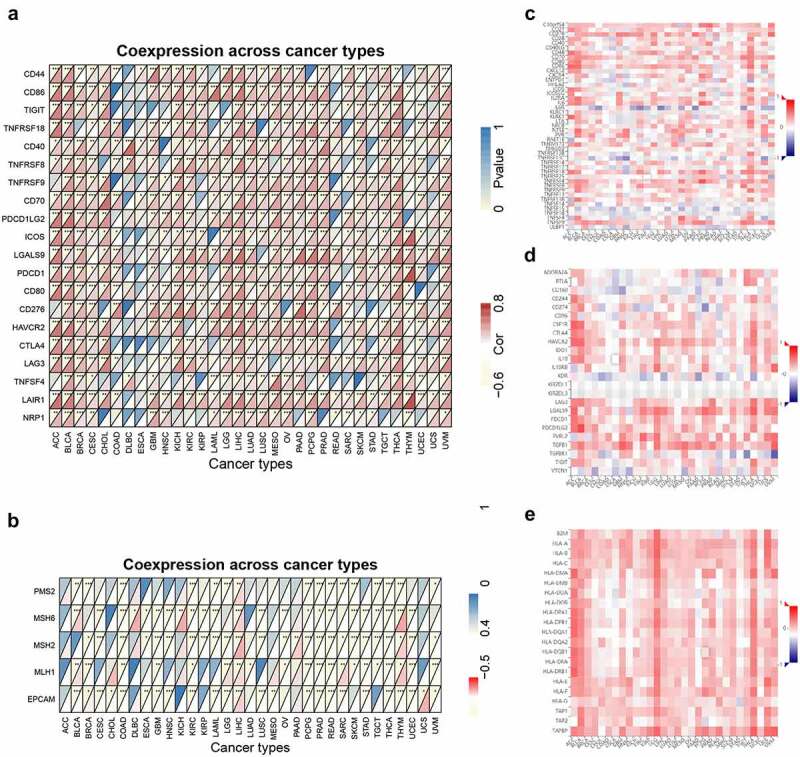
**Correlation analysis of ACTB and immune checkpoints, as well as other signatures across all TCGA cancers**. (a) The correlation of ACTB and known immune checkpoints across all TCGA cancers. (b) The correlation of ACTB and typical MMR signatures across all TCGA cancers. (c) The correlation of ACTB expression and immune stimulators in cancers through tumor-immune system interaction database (TISIDB). (d) The correlation of ACTB expression and immune inhibitors in cancers through TISIDB. (e) The correlation of ACTB expression and MHC molecules in cancers through TISIDB

We next investigated the associations of ACTB and immune checkpoints, immune modulators. In most cancers, except DLBC, ESCA, READ, SARC and UCS, ACTB expression was significantly correlated with the expression levels of known immune checkpoints including lymphocyte-activation gene 3 (LAG3), CD40, cytotoxic T-lymphocyte-associated protein 4 (CTLA4), CD80, programmed cell death 1 (PDCD1), programmed cell death 1 ligand 2 (PDCD1LG2), T cell immunoreceptor with Ig and ITIM domains (TIGIT), CD86 and tumor necrosis factor receptor superfamily, member 9 (TNFRSF9) (Figure 6(a)), indicating a possible cooperation of ACTB with recognized immune checkpoints. Mismatch repair (MMR) system plays an important role in recognizing and repairing mistakes during DNA replication [59]. The relationship of ACTB expression and typical MMR signatures was explored. A positive correlation between ACTB expression and MutL homolog 1 (MLH1), MutS homolog 2 (MSH2), and MutS homolog 6 (MSH6) was shown in BLCA, KICH, KIRP, LIHC, Pancreatic adenocarcinoma (PAAD) and THYM (Figure 6(b)). On the contrary, ACTB expression was negatively correlated with epithelial cell adhesion molecule (EpCAM) in BLCA, CESC, COAD, ESCA, GBM, HNSC, KIRC, LGG, LUSC, PCPG, PRAD, READ, SARC, STAD, THCA, THYM and UCEC.

To further illuminate the relationship of ACTB and immune microenvironment in cancers, we explored the correlations between ACTB and three types of immune modulators expounded in previous study [60] on TISIDB database. The results showed that ACTB expression was positively correlated with most of the immune modulators, including Immune stimulator, Immune inhibitor and major histocompatibility complex (MHC) molecules (Figure 6(c–e)). These results suggested that ACTB might play a crucial role in tumor microenvironment regulation.

Again, stage-specific immune checkpoint analysis was conducted in the 7 cancers with stage dependency in ACTB expression. Most ACTB-immune checkpoint correlations showed stage dependency in one or more cancers (Fig. S5).

### 3.7. Relationship Between ACTB and functional states across different cancer types

**Figure 7.**
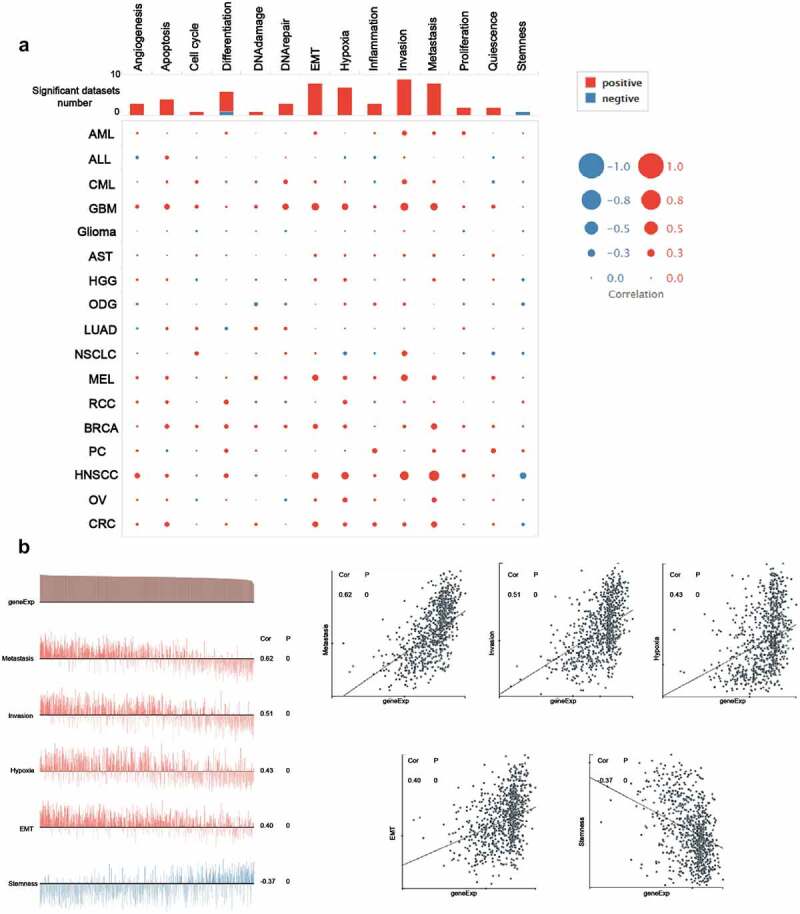
**The functional relevance of ACTB across different cancers from cancerSEA website**. (a) Average correlations between ACTB and functional states in different cancers from cancerSEA. (b) Functional relevance of ACTB in HNSCC from cancerSEA. Red plots suggested a positive correlation while blue plots suggested a negative correlation

To unravel the underlying mechanisms of ACTB in cancers, the correlation between ACTB and functional states of multiple cancers was investigated in CancerSEA database. ACTB was analyzed at single-cell resolution in different cancers (Figure 7(a)). We observed that ACTB was correlated with multiple functional states in multiple cancers, especially in HNSCC. We found ACTB positively correlated with metastasis (ρ = 0.62, *P* = 0), invasion (ρ = 0.51, *P* = 0), hypoxia (ρ = 0.43, *P* = 0.004), epithelial–mesenchymal transition (EMT) (ρ = 0.40, *P* = 0) and negatively correlated with stemness (ρ = -0.37, *P* = 0) in HNSCC (Figure 7(b)). These results reminded us that ACTB might play a quite important role in HNSCC by regulating tumor functions of HNSCC. The detailed information about the functional relevance of ACTB was shown in Table S1.

### 3.8. Enrichment analysis

**Figure 8.**
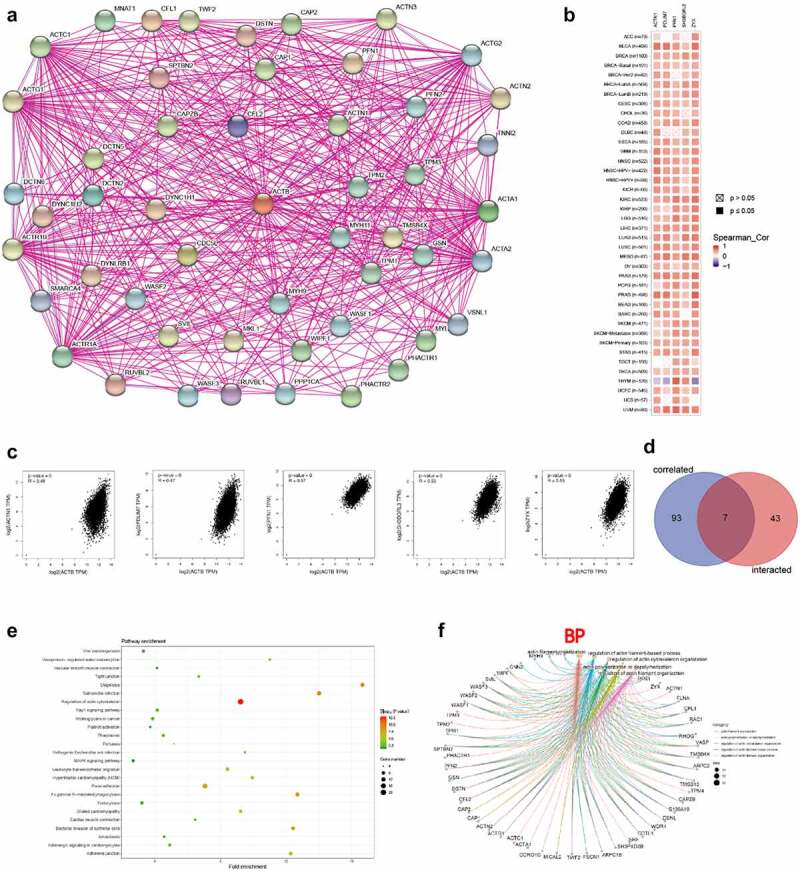
**ACTB-related gene enrichment analysis**. (a) ACTB-interacted proteins from STRING. (b) Heatmap of the expression correlation between ACTB and ACTN1, PDLIM7, PFN1, SH3BGEL3, ZYX in cancers utilizing TIMER2. (c) The expression correlation between ACTB and ACTN1, PDLIM7, PFN1, SH3BGEL3, ZYX utilizing GEPIA2. (d) Venn diagram of the ACTB-interacted and correlated genes. (e) KEGG pathway analysis based on the ACTB-interacted and correlated genes. (f) The cnetplot of the biological process from GO analysis

To further uncover the molecular mechanism of ACTB in oncogenesis and progression, we combined ACTB-binding proteins and ACTB-correlated genes for enrichment analyses. The interaction network of a total of 50 ACTB-binding proteins was acquired using STRING database (Figure 8(a)). In addition, the top 100 ACTB expression-correlated genes were obtained using GEPIA2. ACTB expression was positively correlated with that of the top 5 genes including actinin alpha 1 (ACTN1) (R = 0.48), PDZ and LIM domain 7 (PDLIM7) (R = 0.47), profilin 1 (PFN1) (R = 0.57), SH3 domain binding glutamate-rich protein like 3 (SH3BGRL3) (R = 0.53) and zyxin (ZYX) (R = 0.53) (all P < 0.001) (Figure 8(b-c)). Seven common members of the two groups were screened out as cofilin 1, ACTN1, myosin-heavy chain 9, thymosin beta 4-X-linked, twinfilin actin-binding protein 2, capping protein muscle Z-line-beta and PFN1 (Figure 8(d)). Moreover, KEGG and GO enrichment analyses were conducted for these ACTB-related partners. The KEGG results showed that ‘regulation of actin cytoskeleton’ and ‘focal adhesion’ were involved in the functional mechanism of ACTB in pathogenesis of cancers. The GO enrichment results further suggested that most of ACTB-related genes were associated with actin regulation, such as actin filament organization, actin polymerization and depolymerization, regulation of actin cytoskeleton organization, regulation of actin filament-based process, regulation of actin filament organization (figure 8(f)), and others (Fig. S3).

### 3.9. *The expression and function of ACTB* in vitro

**Figure 9.**
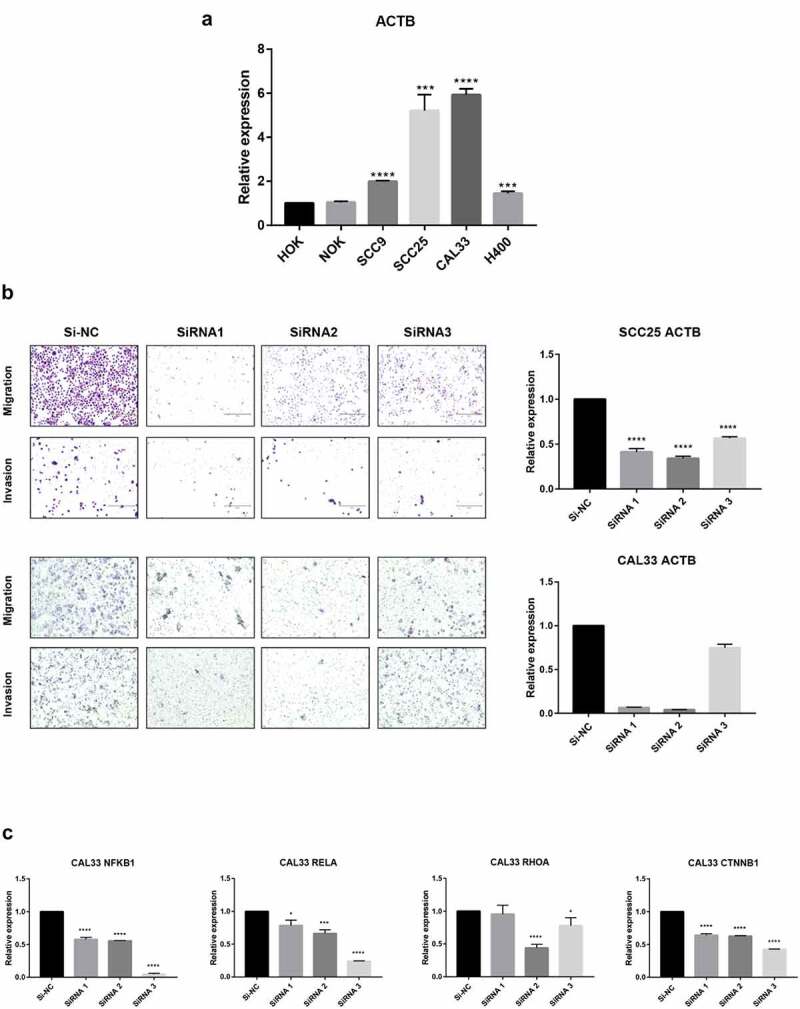
**Validation of ACTB mRNA expression and function in HNSC**. Normal head and neck cell lines include HOK and NOK and HNSC cell lines include SCC9, SCC25, CAL33 and H400. * P < 0.05; ** P < 0.01; ***P < 0.001; ****P < 0.0001. (a) Comparison of ACTB mRNA expression between normal head and neck and HNSC cell lines. (b) Knockdown of ACTB restrained cell migration and invasion in SCC25 and CAL33 cells. (c) Knockdown of ACTB decreased the expression of NFKB1, RELA, RHOA and CTNNB1

In order to further verify the expression of ACTB, we investigated the relative expression of its transcripts in normal head and neck (HOK and NOK) and HNSC (SCC9, SCC25, CAL33 and H400) cell lines by RT-qPCR. The results showed that the ACTB expression was significantly upregulated in HNSC cells SCC9, SCC25, CAL33 and H400 as compared to normal head and neck cells HOK and NOK (Figure 9(a)). Transwell assay showed that deletion of ACTB restrained the migration and invasion of SCC25 and CAL33 cells (Figure 9(b)). Knockdown of ACTB in CAL33 cells resulted in the lower expression of NFKB1, RELA, RHOA and CTNNB1 (β-catenin), indicating that NF-κB and Wnt/β-catenin pathways may take part in ACTB-mediated HNSC development (Figure 9(c)).

## 4. Discussion

Our study indicated that ACTB may play a malignant and complicated role to be reckoned with in tumor development and its role as a housekeep and reference gene in cancer tissues and cell lines should be used with caution. Oncomine and TCGA results demonstrated increased expression of ACTB in multiple cancers like head and neck cancer, leukemia, pancreatic cancer, LIHC, KIRC compared with normal tissues, and ACTB expression was lower in several cancers like lung and prostate cancers. Most of the results were consistent with former studies [15 ~ 19]. However, several studies have found that ACTB was upregulated in lung and prostate cancers [61,62], which need to be validated further in the future. We also found that ACTB expression was higher in stage III and IV compared with stage I and II in several cancers including KICH, KIRC, STAD, TGCT, THCA, ACC and BLCA, indicating ACTB might promote tumor development. Nevertheless, the survival prognosis analysis demonstrated correlation between high ACTB expression and poorer prognosis in different tumors including GBM, HNSC, KIRC, LGG, LIHC, LUAD, MESO, SKCM, UVM and KICH. ACTB is an essential component of cytoskeleton playing a critical role in cell growth and cell migration [5,63], and ACTB was significantly overexpressed in different tumor cell lines of highly invasive ability [9]. The polymerization and remolding of the ACTB could regulate the morphology and phenotype of malignant cells and contribute to tumor malignancy [64–67]. We speculated that ACTB upregulation in cancers might regulate tumor cell proliferation, phenotype and metastasis to further influence the tumor malignancy and prognosis of tumor patients.

Tumor immune infiltrating cells are important roles in tumor immune regulation. More and more studies have suggested that tumor immune infiltrating cells are closely associated with the tumor progression, function of immune checkpoint inhibition and patient prognosis [68–70]. Our immune-related results suggested ACTB plays a specific role in regulating the tumor immune microenvironment and aberrant ACTB expression may alter tumor immune microenvironment. In this study, we first provided evidence of the relationship of ACTB expression and immune infiltration, immune checkpoints, immune regulators or MMR. We observed a significant correlation between ACTB expression and the immune infiltration level of cancer associated fibroblasts, macrophages, endothelial cells, monocytes and CD8 + T-cells in most cancers. It was worth noting that, in THYM, almost all algorithms presented significant but contrary results compared with other cancers, indicating some unique regulatory mechanism may exist in THYM. Additionally, we also found positive correlation between ACTB expression and most immune modulators including many immune checkpoint molecules in pan-cancer, suggesting ACTB was synergistic with checkpoint members and other immune modulators. A few studies have reported that ACTB was upregulated in a proinflammatory phenotype of HCC peritumoral tissues with increased immune infiltration and rBCG-A_N_-E-A_C_ upregulated the ACTB expression in macrophages and enhanced the power of antigen presentation and immune reaction of CD8 + T-cells [71,72]. Considering current limited understanding of ACTB immunological relevance in cancers, our study first presented a comprehensive landscape of immune correlation of ACTB in cancers and we inferred that ACTB might be a potential target of immune therapy. However, further verification is needed.

Nevertheless, we combined ACTB-interacted proteins and ACTB-correlated genes in cancers for enrichment analysis and the latent function of “focal adhesion“, ”regulation of actin cytoskeleton” and “regulation of actin filament” in the pathogenesis of cancers was uncovered. ScRNA-seq analysis from CancerSEA revealed functional relevance of ACTB with metastasis, invasion, hypoxia, differentiation and EMT in different cancers, especially in HNSCC. Current evidence have indicated that ACTB polymerization, cytoskeleton formation and localization could drive cancer cell protrusions and motility, promoting cancer metastasis and invasiveness [5,17,19,73]. ACTB was found involved in EMT-related signaling pathway in RCC and renal fibrosis [74,75], and ACTB-MITF gene fusion could identify melanocytic differentiation of clear cell cutaneous neoplasms [76]. Further experimental validation in HNSC confirmed that ACTB was significantly overexpressed in HNSC cell lines compared with normal head and neck cell lines. Previous studies have reported that ACTB expression was higher in multiple cancers, like HCC, RCC, GC, PC and NSCLC, compared with normal samples [20,61,73,77]. However, in several studies, ACTB was found lower in cell lines of CRC and BC [78,79]. We speculated that it might resulted from different tumor stage or regulation of other molecules like microRNAs in these cancers. We also verified ACTB functions in HNSC and found knockdown of ACTB restrained HNSC cell migration and invasion by regulating the expression of NFKB1, RELA, RHOA and CTNNB1. NFKB1 and RELA are biomarkers in NF-κB pathway, which has been reported to regulate tumor invasiveness [80]. CTNNB2(β-catenin) and RHOA in Wnt/β-catenin were related to focal adhesion and regulated tumor metastasis and invasion [81,82]. ACTB might regulate tumor metastasis and invasion by NF-κB and Wnt/β-catenin pathways in HNSC and other cancers, while further validation in different cancers is needed.

As the development of online database and application of bioinformatics, more and more molecules have been revealed to be involved in tumor development. Solute carrier family 12 member 8 has been reported to be upregulated in bladder cancer and related to patient prognosis and tumor immune cell infiltration [83]. C-X-C chemokine receptor type 4 was identified as a therapeutic target of triptolide in the treatment of STAD patients by modulating the TME [84]. Domain-containing protein 1 has been reported upregulated in most cancers and played oncogenic roles in pan-cancer by comprehensive bioinformatic analysis [85]. Ras-related Protein Rap1b expression was corelated with poor prognosis and tumor immune infiltration in pan-cancer [86]. A 12-gene signature related to iron metabolism was developed in lung cancer [87]. NOP2 expression was found to be related to stage, age, grade and prognosis in RCC [88]. HSF1 was observed upregulated in multiple cancers and might be a potential target for immunotherapy [89]. Compared with former studies, our study challenged the common role of ACTB as a reference gene and verified its role in cancers by different databases, algorithms and experiments.

There are several limitations in our study. More experimental verification is wanted, for example by immunohistochemistry, and immunocytochemistry. And further exploration of related molecular regulatory mechanism in different cancers is wanted.

## 5. Conclusion

In conclusion, our first pan-cancer analyses of ACTB presented statistically significant correlations of ACTB expression with patient prognosis, immune cell infiltration, immune checkpoints, other immune modulators and functional states across different cancers. Our study aids in understanding the role of ACTB in tumorigenesis, presenting a potential prognosis biomarker and immunotherapy target.

## Supplementary Material

Supplemental Material

## Data Availability

All data in our study are available upon reasonable request.
