# Supplementary material for: A pan-cancer analysis of the prognostic and immunological role of β-actin (ACTB) in human cancers
Source: Bioengineered. 2021 Sep 4;12(1):6166–85. doi: 10.1080/21655979.2021.1973220 (PMC8806805; doi:10.1080/21655979.2021.1973220)
Supplement: Supplemental Material [file KBIE_A_1973220_SM3325.zip › supplementary/Supplementary Materials.docx]

**Supplementary Materials**

Figure S1: Survival analysis using the Kaplan-Meier plotter

Figure S2: Survival analysis based on GEO datasets using prognoscan

Figure S3: Stage-specific OS analysis

Figure S4: Stage- specific immune infiltration analysis

Figure S5: Stage- specific immune checkpoint analysis

Figure S6: The GO enrichment analysis of ACTB-related partners

Table S1: The detailed information about the functional relevance of ACTB
