# Supplementary material for: A pan-cancer analysis of the prognostic and immunological role of β-actin (ACTB) in human cancers
Source: Bioengineered. 2021 Sep 4;12(1):6166–85. doi: 10.1080/21655979.2021.1973220 (PMC8806805; doi:10.1080/21655979.2021.1973220)
Supplement: Supplemental Material [file KBIE_A_1973220_SM3325.zip › supplementary/Table S1.pdf]

Table-S1. **The detailed information about the functional relevance of ACTB**

ExpID	Cancer	State	Correlation	Pvalue
EXP0046	ALL	Angiogenesis	-0.165	0.031
EXP0046	ALL	Apoptosis	0.207	0.006
EXP0046	ALL	CellCycle	0.052	0.497
EXP0046	ALL	Differentiation	0.007	0.926
EXP0046	ALL	DNAdamage	0.008	0.92
EXP0046	ALL	DNArepair	0.044	0.57
EXP0046	ALL	EMT	-0.016	0.835
EXP0046	ALL	Hypoxia	-0.107	0.162
EXP0046	ALL	Inflammation	-0.142	0.063
EXP0046	ALL	Invasion	0.085	0.268
EXP0046	ALL	Metastasis	0.007	0.926
EXP0046	ALL	Proliferation	-0.018	0.813
EXP0046	ALL	Quiescence	-0.105	0.172
EXP0046	ALL	Stemness	0.044	0.568
EXP0047	AML	Angiogenesis	0.366	0
EXP0047	AML	Apoptosis	0.132	0
EXP0047	AML	CellCycle	0.129	0
EXP0047	AML	Differentiation	0.367	0
EXP0047	AML	DNAdamage	0.101	0.001
EXP0047	AML	DNArepair	-0.003	0.909
EXP0047	AML	EMT	0.3	0
EXP0047	AML	Hypoxia	0.049	0.1
EXP0047	AML	Inflammation	0.353	0
EXP0047	AML	Invasion	0.413	0
EXP0047	AML	Metastasis	0.452	0
EXP0047	AML	Proliferation	0.402	0
EXP0047	AML	Quiescence	0.198	0
EXP0047	AML	Stemness	-0.012	0.7
EXP0048	AML	Angiogenesis	-0.108	0.166
EXP0048	AML	Apoptosis	-0.128	0.102
EXP0048	AML	CellCycle	0.073	0.351
EXP0048	AML	Differentiation	-0.14	0.074
EXP0048	AML	DNAdamage	-0.023	0.772
EXP0048	AML	DNArepair	0.246	0.001
EXP0048	AML	EMT	0.206	0.008
EXP0048	AML	Hypoxia	-0.287	0
EXP0048	AML	Inflammation	-0.137	0.08
EXP0048	AML	Invasion	0.342	0
EXP0048	AML	Metastasis	0.061	0.434
EXP0048	AML	Proliferation	-0.031	0.69
EXP0048	AML	Quiescence	-0.299	0

EXP0048	AML	Stemness	0.139	0.076
EXP0049	AML	Angiogenesis	0.007	0.949
EXP0049	AML	Apoptosis	0.058	0.573
EXP0049	AML	CellCycle	-0.17	0.099
EXP0049	AML	Differentiation	0.156	0.13
EXP0049	AML	DNAdamage	-0.113	0.273
EXP0049	AML	DNArepair	-0.175	0.088
EXP0049	AML	EMT	-0.022	0.83
EXP0049	AML	Hypoxia	0.219	0.032
EXP0049	AML	Inflammation	0.061	0.554
EXP0049	AML	Invasion	0.068	0.511
EXP0049	AML	Metastasis	0.022	0.834
EXP0049	AML	Proliferation	0.216	0.034
EXP0049	AML	Quiescence	0.094	0.364
EXP0049	AML	Stemness	-0.286	0.005
EXP0050	CML	Angiogenesis	-0.047	0.043
EXP0050	CML	Apoptosis	0.119	0
EXP0050	CML	CellCycle	0.186	0
EXP0050	CML	Differentiation	0.097	0
EXP0050	CML	DNAdamage	0.107	0
EXP0050	CML	DNArepair	0.247	0
EXP0050	CML	EMT	0.143	0
EXP0050	CML	Hypoxia	0.085	0
EXP0050	CML	Inflammation	-0.112	0
EXP0050	CML	Invasion	0.276	0
EXP0050	CML	Metastasis	0.144	0
EXP0050	CML	Proliferation	0.044	0.057
EXP0050	CML	Quiescence	-0.15	0
EXP0050	CML	Stemness	-0.081	0
EXP0051	CRC	Angiogenesis	0.131	0.027
EXP0051	CRC	Apoptosis	0.27	0
EXP0051	CRC	CellCycle	0.023	0.696
EXP0051	CRC	Differentiation	0.113	0.056
EXP0051	CRC	DNAdamage	0.152	0.01
EXP0051	CRC	DNArepair	-0.011	0.852
EXP0051	CRC	EMT	0.32	0
EXP0051	CRC	Hypoxia	0.216	0
EXP0051	CRC	Inflammation	0.229	0
EXP0051	CRC	Invasion	0.223	0
EXP0051	CRC	Metastasis	0.34	0
EXP0051	CRC	Proliferation	0.051	0.395
EXP0051	CRC	Quiescence	0.126	0.033
EXP0051	CRC	Stemness	-0.174	0.003

EXP0052	BRCA	Angiogenesis	-0.025	0.638
EXP0052	BRCA	Apoptosis	0.156	0.003
EXP0052	BRCA	CellCycle	0.087	0.097
EXP0052	BRCA	Differentiation	-0.078	0.135
EXP0052	BRCA	DNAdamage	0.229	0
EXP0052	BRCA	DNArepair	0.117	0.025
EXP0052	BRCA	EMT	0.112	0.032
EXP0052	BRCA	Hypoxia	0.17	0.001
EXP0052	BRCA	Inflammation	-0.244	0
EXP0052	BRCA	Invasion	0.085	0.102
EXP0052	BRCA	Metastasis	0.276	0
EXP0052	BRCA	Proliferation	-0.053	0.314
EXP0052	BRCA	Quiescence	-0.114	0.029
EXP0052	BRCA	Stemness	0.084	0.105
EXP0053	BRCA	Angiogenesis	-0.003	0.956
EXP0053	BRCA	Apoptosis	0.324	0
EXP0053	BRCA	CellCycle	0.066	0.245
EXP0053	BRCA	Differentiation	0.158	0.005
EXP0053	BRCA	DNAdamage	0.239	0
EXP0053	BRCA	DNArepair	0.21	0
EXP0053	BRCA	EMT	0.098	0.081
EXP0053	BRCA	Hypoxia	0.316	0
EXP0053	BRCA	Inflammation	0.153	0.006
EXP0053	BRCA	Invasion	0.231	0
EXP0053	BRCA	Metastasis	0.169	0.003
EXP0053	BRCA	Proliferation	0.051	0.369
EXP0053	BRCA	Quiescence	0.224	0
EXP0053	BRCA	Stemness	0.169	0.002
EXP0054	BRCA	Angiogenesis	0.157	0.195
EXP0054	BRCA	Apoptosis	0.471	0
EXP0054	BRCA	CellCycle	0.361	0.002
EXP0054	BRCA	Differentiation	0.521	0
EXP0054	BRCA	DNAdamage	0.417	0
EXP0054	BRCA	DNArepair	0.313	0.008
EXP0054	BRCA	EMT	0.333	0.005
EXP0054	BRCA	Hypoxia	0.531	0
EXP0054	BRCA	Inflammation	0.364	0.002
EXP0054	BRCA	Invasion	0.506	0
EXP0054	BRCA	Metastasis	0.525	0
EXP0054	BRCA	Proliferation	0.408	0
EXP0054	BRCA	Quiescence	0.424	0
EXP0054	BRCA	Stemness	0.055	0.651
EXP0055	BRCA	Angiogenesis	0.191	0.295

EXP0055	BRCA	Apoptosis	0.029	0.877
EXP0055	BRCA	CellCycle	0.186	0.307
EXP0055	BRCA	Differentiation	0.355	0.047
EXP0055	BRCA	DNAdamage	-0.253	0.161
EXP0055	BRCA	DNArepair	0.106	0.565
EXP0055	BRCA	EMT	0.561	0.001
EXP0055	BRCA	Hypoxia	-0.21	0.248
EXP0055	BRCA	Inflammation	-0.081	0.657
EXP0055	BRCA	Invasion	-0.091	0.621
EXP0055	BRCA	Metastasis	0.426	0.016
EXP0055	BRCA	Proliferation	0.168	0.359
EXP0055	BRCA	Quiescence	0.085	0.642
EXP0055	BRCA	Stemness	0.047	0.798
EXP0056	AST	Angiogenesis	0.067	0
EXP0056	AST	Apoptosis	0.028	0.048
EXP0056	AST	CellCycle	0.095	0
EXP0056	AST	Differentiation	0.059	0
EXP0056	AST	DNAdamage	0.016	0.241
EXP0056	AST	DNArepair	-0.007	0.631
EXP0056	AST	EMT	0.147	0
EXP0056	AST	Hypoxia	0.117	0
EXP0056	AST	Inflammation	0.103	0
EXP0056	AST	Invasion	0.127	0
EXP0056	AST	Metastasis	0.147	0
EXP0056	AST	Proliferation	0.048	0.001
EXP0056	AST	Quiescence	0.137	0
EXP0056	AST	Stemness	0	0.973
EXP0057	GBM	Angiogenesis	0.372	0
EXP0057	GBM	Apoptosis	0.356	0
EXP0057	GBM	CellCycle	0.194	0
EXP0057	GBM	Differentiation	0.214	0
EXP0057	GBM	DNAdamage	0.105	0.001
EXP0057	GBM	DNArepair	0.301	0
EXP0057	GBM	EMT	0.513	0
EXP0057	GBM	Hypoxia	0.388	0
EXP0057	GBM	Inflammation	0.244	0
EXP0057	GBM	Invasion	0.547	0
EXP0057	GBM	Metastasis	0.515	0
EXP0057	GBM	Proliferation	0.177	0
EXP0057	GBM	Quiescence	0.256	0
EXP0057	GBM	Stemness	-0.135	0
EXP0058	GBM	Angiogenesis	0.03	0.459
EXP0058	GBM	Apoptosis	0.277	0

EXP0058	GBM	CellCycle	0.215	0
EXP0058	GBM	Differentiation	-0.021	0.601
EXP0058	GBM	DNAdamage	0.249	0
EXP0058	GBM	DNArepair	0.408	0
EXP0058	GBM	EMT	0.345	0
EXP0058	GBM	Hypoxia	0.379	0
EXP0058	GBM	Inflammation	0.047	0.237
EXP0058	GBM	Invasion	0.364	0
EXP0058	GBM	Metastasis	0.337	0
EXP0058	GBM	Proliferation	0.076	0.058
EXP0058	GBM	Quiescence	0.16	0
EXP0058	GBM	Stemness	0.062	0.124
EXP0059	Glioma	Angiogenesis	0.031	0.143
EXP0059	Glioma	Apoptosis	0.012	0.565
EXP0059	Glioma	CellCycle	-0.128	0
EXP0059	Glioma	Differentiation	0.001	0.956
EXP0059	Glioma	DNAdamage	-0.045	0.034
EXP0059	Glioma	DNArepair	-0.075	0
EXP0059	Glioma	EMT	-0.074	0
EXP0059	Glioma	Hypoxia	0.06	0.004
EXP0059	Glioma	Inflammation	0.014	0.514
EXP0059	Glioma	Invasion	0.071	0.001
EXP0059	Glioma	Metastasis	-0.021	0.32
EXP0059	Glioma	Proliferation	-0.115	0
EXP0059	Glioma	Quiescence	0.094	0
EXP0059	Glioma	Stemness	-0.059	0.005
EXP0060	Glioma	Angiogenesis	-0.04	0.608
EXP0060	Glioma	Apoptosis	0.066	0.393
EXP0060	Glioma	CellCycle	-0.063	0.419
EXP0060	Glioma	Differentiation	-0.131	0.092
EXP0060	Glioma	DNAdamage	-0.053	0.497
EXP0060	Glioma	DNArepair	-0.12	0.123
EXP0060	Glioma	EMT	0.053	0.496
EXP0060	Glioma	Hypoxia	-0.134	0.084
EXP0060	Glioma	Inflammation	-0.102	0.19
EXP0060	Glioma	Invasion	0.071	0.36
EXP0060	Glioma	Metastasis	-0.052	0.507
EXP0060	Glioma	Proliferation	-0.096	0.216
EXP0060	Glioma	Quiescence	-0.081	0.301
EXP0060	Glioma	Stemness	-0.101	0.193
EXP0061	HGG	Angiogenesis	0.103	0
EXP0061	HGG	Apoptosis	0.113	0
EXP0061	HGG	CellCycle	-0.118	0

EXP0061	HGG	Differentiation	0.033	0.084
EXP0061	HGG	DNAdamage	-0.1	0
EXP0061	HGG	DNArepair	-0.071	0
EXP0061	HGG	EMT	0.144	0
EXP0061	HGG	Hypoxia	0.172	0
EXP0061	HGG	Inflammation	0.047	0.014
EXP0061	HGG	Invasion	0.089	0
EXP0061	HGG	Metastasis	0.16	0
EXP0061	HGG	Proliferation	-0.106	0
EXP0061	HGG	Quiescence	0.103	0
EXP0061	HGG	Stemness	-0.161	0
EXP0062	ODG	Angiogenesis	-0.108	0
EXP0062	ODG	Apoptosis	0.019	0.235
EXP0062	ODG	CellCycle	-0.003	0.862
EXP0062	ODG	Differentiation	-0.029	0.061
EXP0062	ODG	DNAdamage	-0.208	0
EXP0062	ODG	DNArepair	-0.129	0
EXP0062	ODG	EMT	0.014	0.376
EXP0062	ODG	Hypoxia	0.136	0
EXP0062	ODG	Inflammation	0.16	0
EXP0062	ODG	Invasion	0.157	0
EXP0062	ODG	Metastasis	-0.008	0.617
EXP0062	ODG	Proliferation	-0.101	0
EXP0062	ODG	Quiescence	0.074	0
EXP0062	ODG	Stemness	-0.186	0
EXP0063	HNSCC	Angiogenesis	0.298	0
EXP0063	HNSCC	Apoptosis	0.198	0
EXP0063	HNSCC	CellCycle	0.075	0.001
EXP0063	HNSCC	Differentiation	0.267	0
EXP0063	HNSCC	DNAdamage	0.105	0
EXP0063	HNSCC	DNArepair	-0.003	0.889
EXP0063	HNSCC	EMT	0.395	0
EXP0063	HNSCC	Hypoxia	0.427	0
EXP0063	HNSCC	Inflammation	0.151	0
EXP0063	HNSCC	Invasion	0.512	0
EXP0063	HNSCC	Metastasis	0.62	0
EXP0063	HNSCC	Proliferation	0.198	0
EXP0063	HNSCC	Quiescence	0.126	0
EXP0063	HNSCC	Stemness	-0.369	0
EXP0064	RCC	Angiogenesis	0.222	0.043
EXP0064	RCC	Apoptosis	0.296	0.007
EXP0064	RCC	CellCycle	-0.041	0.715
EXP0064	RCC	Differentiation	0.382	0

EXP0064	RCC	DNAdamage	-0.049	0.662
EXP0064	RCC	DNArepair	-0.074	0.503
EXP0064	RCC	EMT	-0.033	0.764
EXP0064	RCC	Hypoxia	0.322	0.003
EXP0064	RCC	Inflammation	-0.115	0.301
EXP0064	RCC	Invasion	0.106	0.338
EXP0064	RCC	Metastasis	0.176	0.111
EXP0064	RCC	Proliferation	-0.002	0.986
EXP0064	RCC	Quiescence	-0.024	0.831
EXP0064	RCC	Stemness	0.24	0.029
EXP0065	RCC	Angiogenesis	0.029	0.867
EXP0065	RCC	Apoptosis	0.084	0.631
EXP0065	RCC	CellCycle	-0.031	0.858
EXP0065	RCC	Differentiation	0.155	0.375
EXP0065	RCC	DNAdamage	-0.177	0.309
EXP0065	RCC	DNArepair	-0.15	0.389
EXP0065	RCC	EMT	0.094	0.591
EXP0065	RCC	Hypoxia	0.152	0.383
EXP0065	RCC	Inflammation	-0.096	0.585
EXP0065	RCC	Invasion	0.121	0.488
EXP0065	RCC	Metastasis	-0.011	0.949
EXP0065	RCC	Proliferation	-0.015	0.934
EXP0065	RCC	Quiescence	-0.054	0.756
EXP0065	RCC	Stemness	0.014	0.937
EXP0066	LUAD	Angiogenesis	-0.115	0.2
EXP0066	LUAD	Apoptosis	0.275	0.002
EXP0066	LUAD	CellCycle	0.154	0.086
EXP0066	LUAD	Differentiation	-0.043	0.633
EXP0066	LUAD	DNAdamage	0.141	0.116
EXP0066	LUAD	DNArepair	0.193	0.03
EXP0066	LUAD	EMT	0.022	0.81
EXP0066	LUAD	Hypoxia	0.107	0.233
EXP0066	LUAD	Inflammation	0.014	0.877
EXP0066	LUAD	Invasion	0.2	0.025
EXP0066	LUAD	Metastasis	-0.008	0.933
EXP0066	LUAD	Proliferation	0.138	0.123
EXP0066	LUAD	Quiescence	-0.065	0.471
EXP0066	LUAD	Stemness	0.031	0.732
EXP0067	LUAD	Angiogenesis	-0.063	0.692
EXP0067	LUAD	Apoptosis	0.062	0.697
EXP0067	LUAD	CellCycle	0.191	0.227
EXP0067	LUAD	Differentiation	-0.305	0.05
EXP0067	LUAD	DNAdamage	0.199	0.207

EXP0067	LUAD	DNArepair	0.173	0.272
EXP0067	LUAD	EMT	-0.03	0.852
EXP0067	LUAD	Hypoxia	-0.03	0.851
EXP0067	LUAD	Inflammation	0.012	0.938
EXP0067	LUAD	Invasion	-0.093	0.559
EXP0067	LUAD	Metastasis	0.046	0.773
EXP0067	LUAD	Proliferation	0.094	0.553
EXP0067	LUAD	Quiescence	0.031	0.848
EXP0067	LUAD	Stemness	-0.083	0.601
EXP0068	NSCLC	Angiogenesis	-0.052	0.002
EXP0068	NSCLC	Apoptosis	0.006	0.704
EXP0068	NSCLC	CellCycle	0.23	0
EXP0068	NSCLC	Differentiation	0.004	0.83
EXP0068	NSCLC	DNAdamage	0.064	0
EXP0068	NSCLC	DNArepair	0.124	0
EXP0068	NSCLC	EMT	0.098	0
EXP0068	NSCLC	Hypoxia	-0.194	0
EXP0068	NSCLC	Inflammation	-0.098	0
EXP0068	NSCLC	Invasion	0.305	0
EXP0068	NSCLC	Metastasis	0.003	0.875
EXP0068	NSCLC	Proliferation	0.096	0
EXP0068	NSCLC	Quiescence	-0.187	0
EXP0068	NSCLC	Stemness	-0.144	0
EXP0069	OV	Angiogenesis	0.083	0.199
EXP0069	OV	Apoptosis	0.106	0.098
EXP0069	OV	CellCycle	-0.111	0.083
EXP0069	OV	Differentiation	0.066	0.302
EXP0069	OV	DNAdamage	-0.012	0.855
EXP0069	OV	DNArepair	-0.13	0.043
EXP0069	OV	EMT	0.15	0.019
EXP0069	OV	Hypoxia	0.271	0
EXP0069	OV	Inflammation	0.122	0.058
EXP0069	OV	Invasion	-0.05	0.439
EXP0069	OV	Metastasis	0.277	0
EXP0069	OV	Proliferation	0.057	0.378
EXP0069	OV	Quiescence	0.092	0.153
EXP0069	OV	Stemness	-0.08	0.215
EXP0070	PC	Angiogenesis	0.107	0.252
EXP0070	PC	Apoptosis	-0.131	0.158
EXP0070	PC	CellCycle	-0.053	0.571
EXP0070	PC	Differentiation	0.225	0.015
EXP0070	PC	DNAdamage	0.084	0.37
EXP0070	PC	DNArepair	-0.037	0.689

EXP0070	PC	EMT	0.006	0.949
EXP0070	PC	Hypoxia	0.049	0.603
EXP0070	PC	Inflammation	0.279	0.002
EXP0070	PC	Invasion	-0.052	0.579
EXP0070	PC	Metastasis	0.198	0.032
EXP0070	PC	Proliferation	0.164	0.078
EXP0070	PC	Quiescence	0.274	0.003
EXP0070	PC	Stemness	0.151	0.104
EXP0071	MEL	Angiogenesis	-0.114	0
EXP0071	MEL	Apoptosis	0.03	0.293
EXP0071	MEL	CellCycle	0.126	0
EXP0071	MEL	Differentiation	-0.162	0
EXP0071	MEL	DNAdamage	0.241	0
EXP0071	MEL	DNArepair	0.183	0
EXP0071	MEL	EMT	0.087	0.002
EXP0071	MEL	Hypoxia	0.046	0.105
EXP0071	MEL	Inflammation	-0.156	0
EXP0071	MEL	Invasion	0.356	0
EXP0071	MEL	Metastasis	-0.174	0
EXP0071	MEL	Proliferation	-0.017	0.546
EXP0071	MEL	Quiescence	-0.067	0.017
EXP0071	MEL	Stemness	-0.108	0
EXP0072	MEL	Angiogenesis	0.398	0
EXP0072	MEL	Apoptosis	0.353	0
EXP0072	MEL	CellCycle	0.013	0.826
EXP0072	MEL	Differentiation	0.352	0
EXP0072	MEL	DNAdamage	0.166	0.003
EXP0072	MEL	DNArepair	0.144	0.012
EXP0072	MEL	EMT	0.6	0
EXP0072	MEL	Hypoxia	0.401	0
EXP0072	MEL	Inflammation	0.475	0
EXP0072	MEL	Invasion	0.405	0
EXP0072	MEL	Metastasis	0.681	0
EXP0072	MEL	Proliferation	-0.058	0.315
EXP0072	MEL	Quiescence	0.47	0
EXP0072	MEL	Stemness	-0.012	0.828
