# Supplementary figures and images for: A pan-cancer analysis of the prognostic and immunological role of β-actin (ACTB) in human cancers
Source: Bioengineered. 2021 Sep 4;12(1):6166–85. doi: 10.1080/21655979.2021.1973220 (PMC8806805; doi:10.1080/21655979.2021.1973220)

A

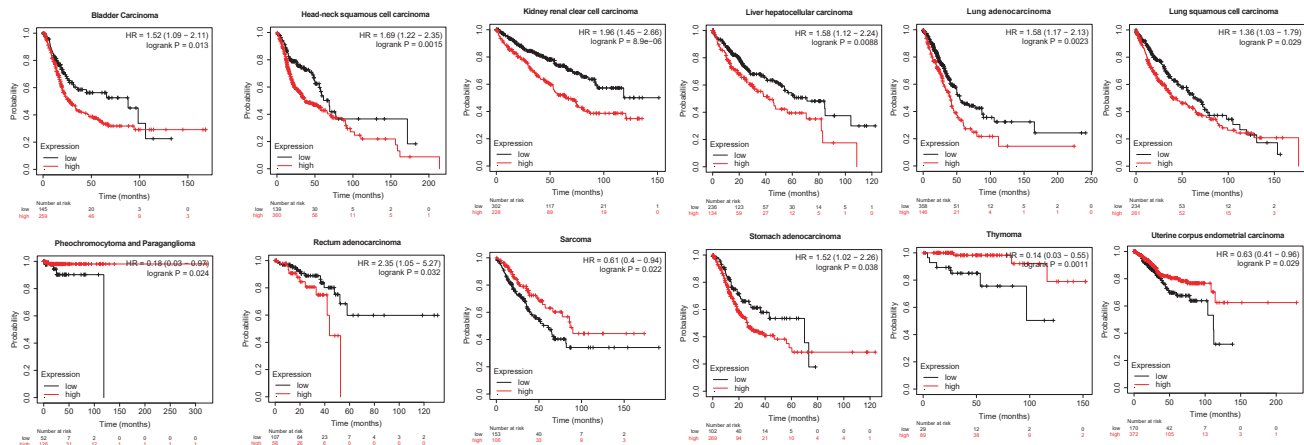

B

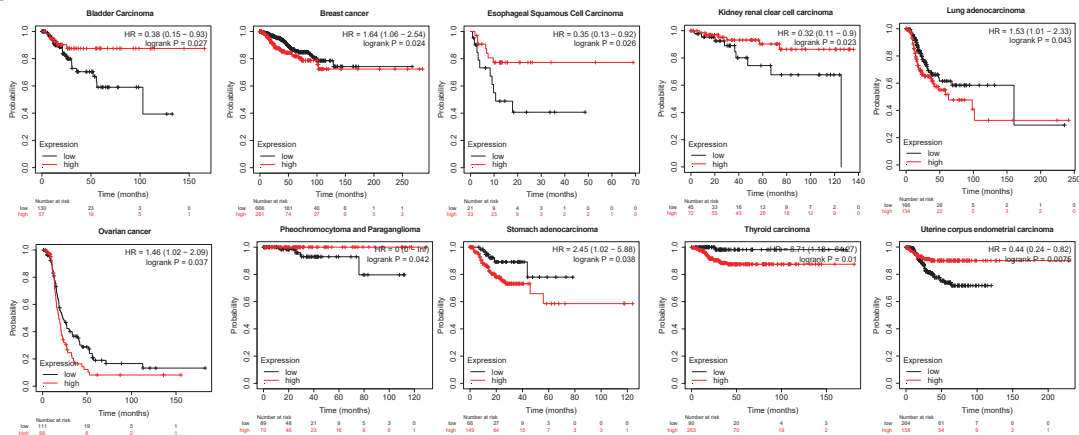

Supplement: Supplemental Material [file KBIE_A_1973220_SM3325.zip › supplementary/Figure S1.pdf]

**A**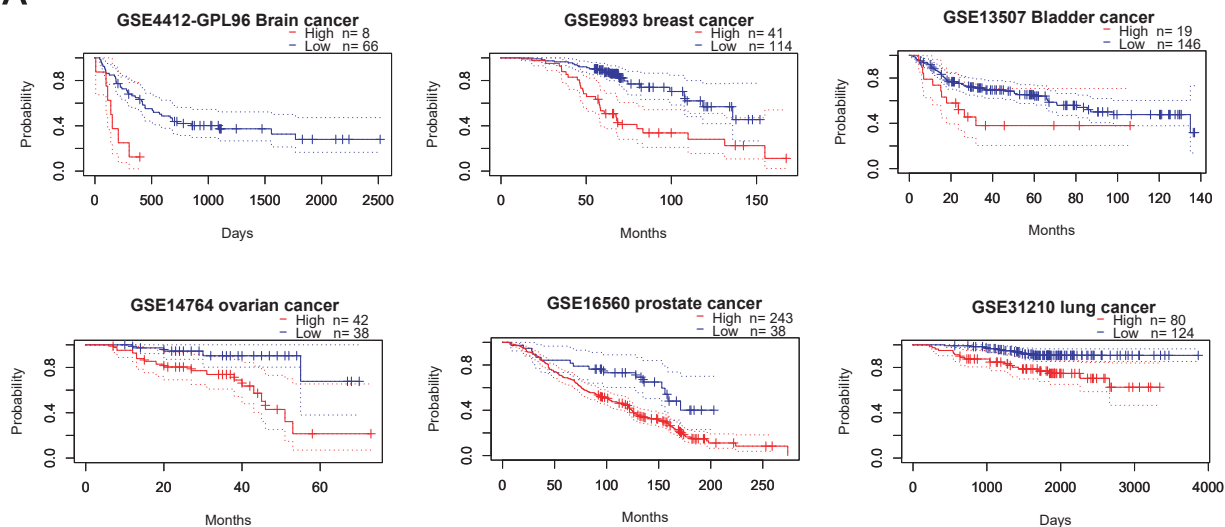**B**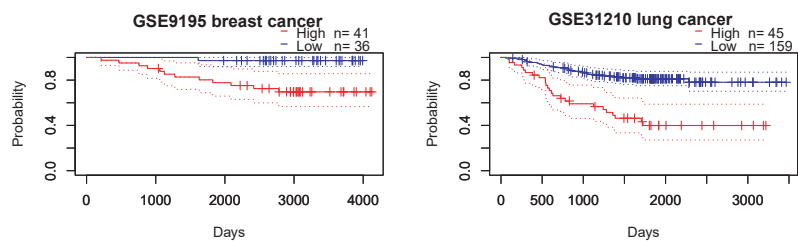**C**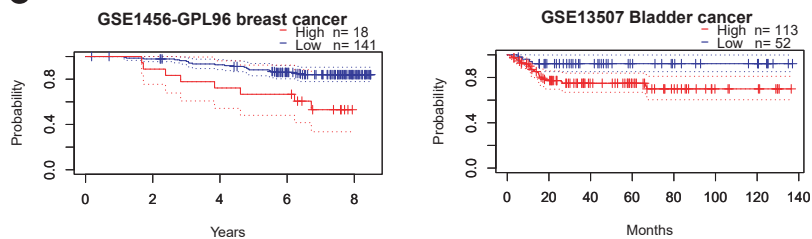**D**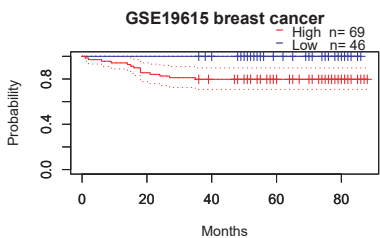**E**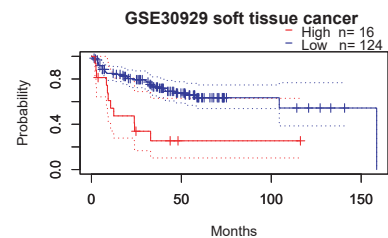**F**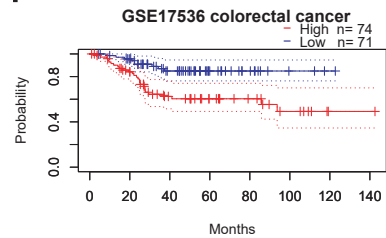

Supplement: Supplemental Material [file KBIE_A_1973220_SM3325.zip › supplementary/Figure S2.pdf]

A

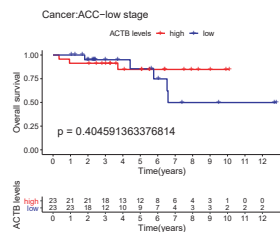

B

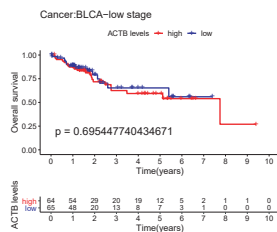

C

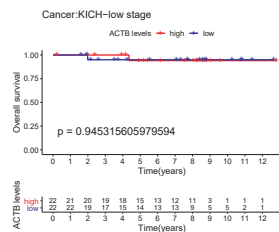

D

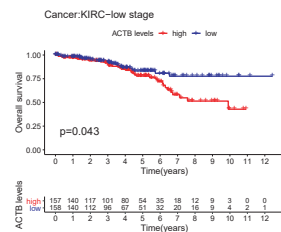

E

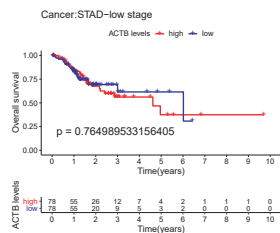

F

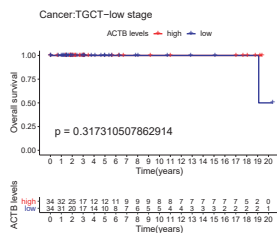

G

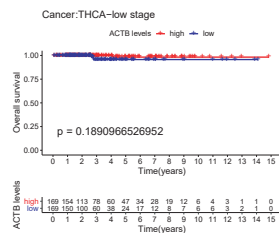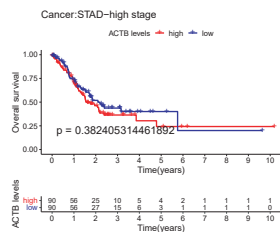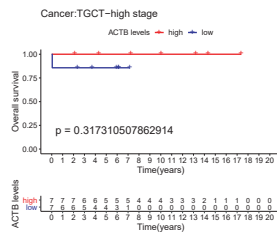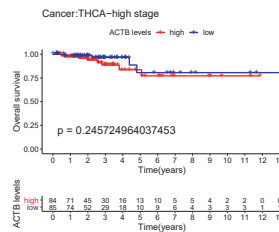

Supplement: Supplemental Material [file KBIE_A_1973220_SM3325.zip › supplementary/Figure S3.pdf]

**A**

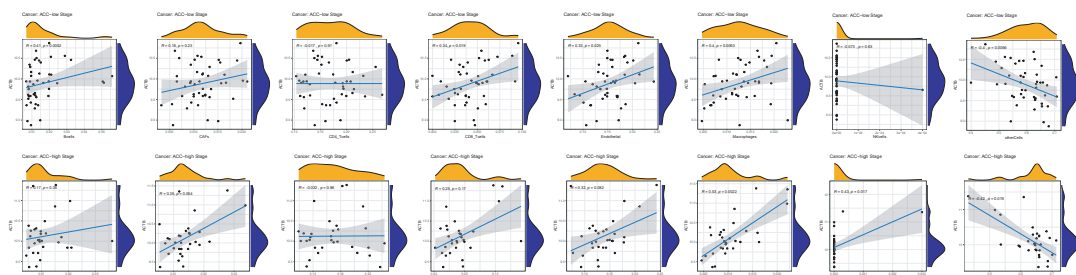

# B

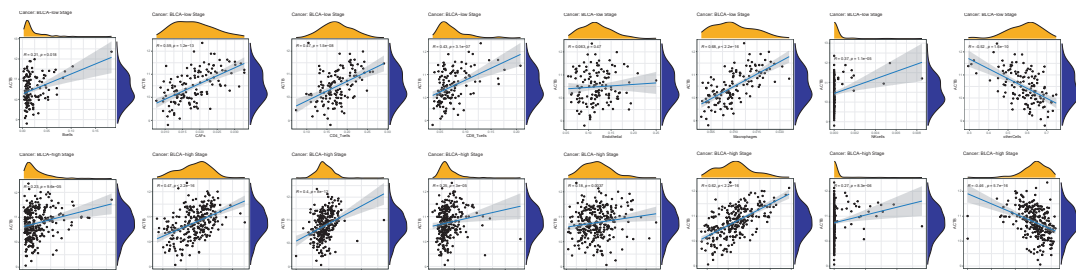

**C**

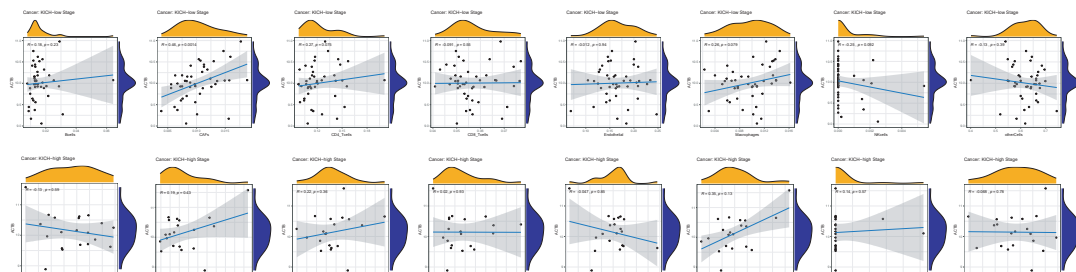

D

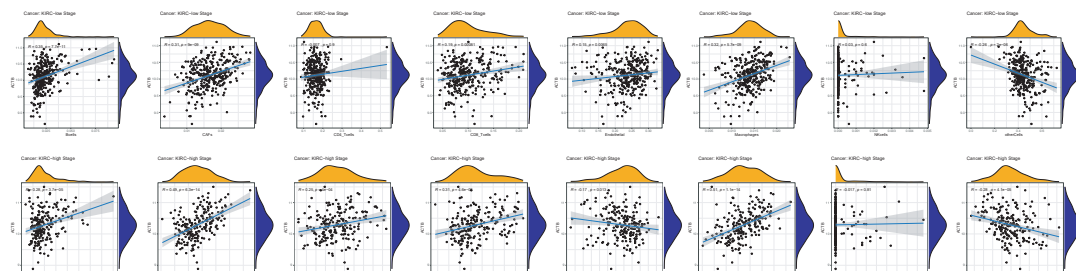

# E

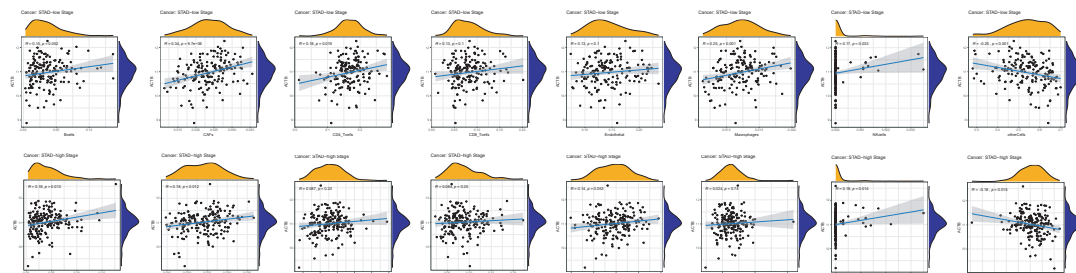

**F**

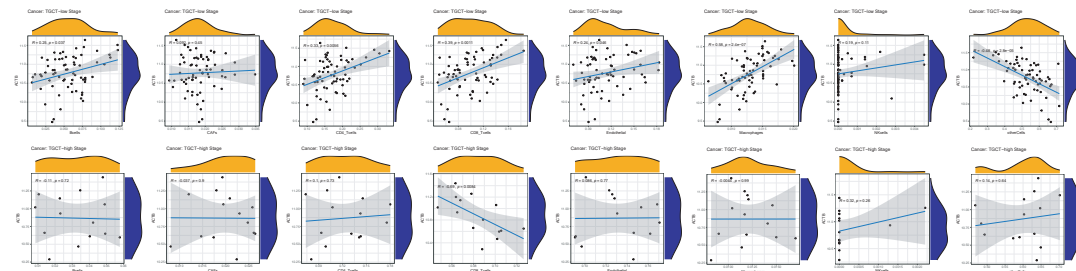

## G

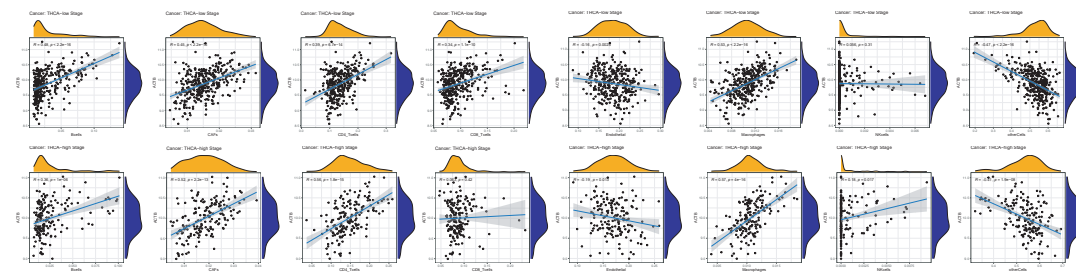

Supplement: Supplemental Material [file KBIE_A_1973220_SM3325.zip › supplementary/Figure S4.pdf]

# Coexpression across cancer types

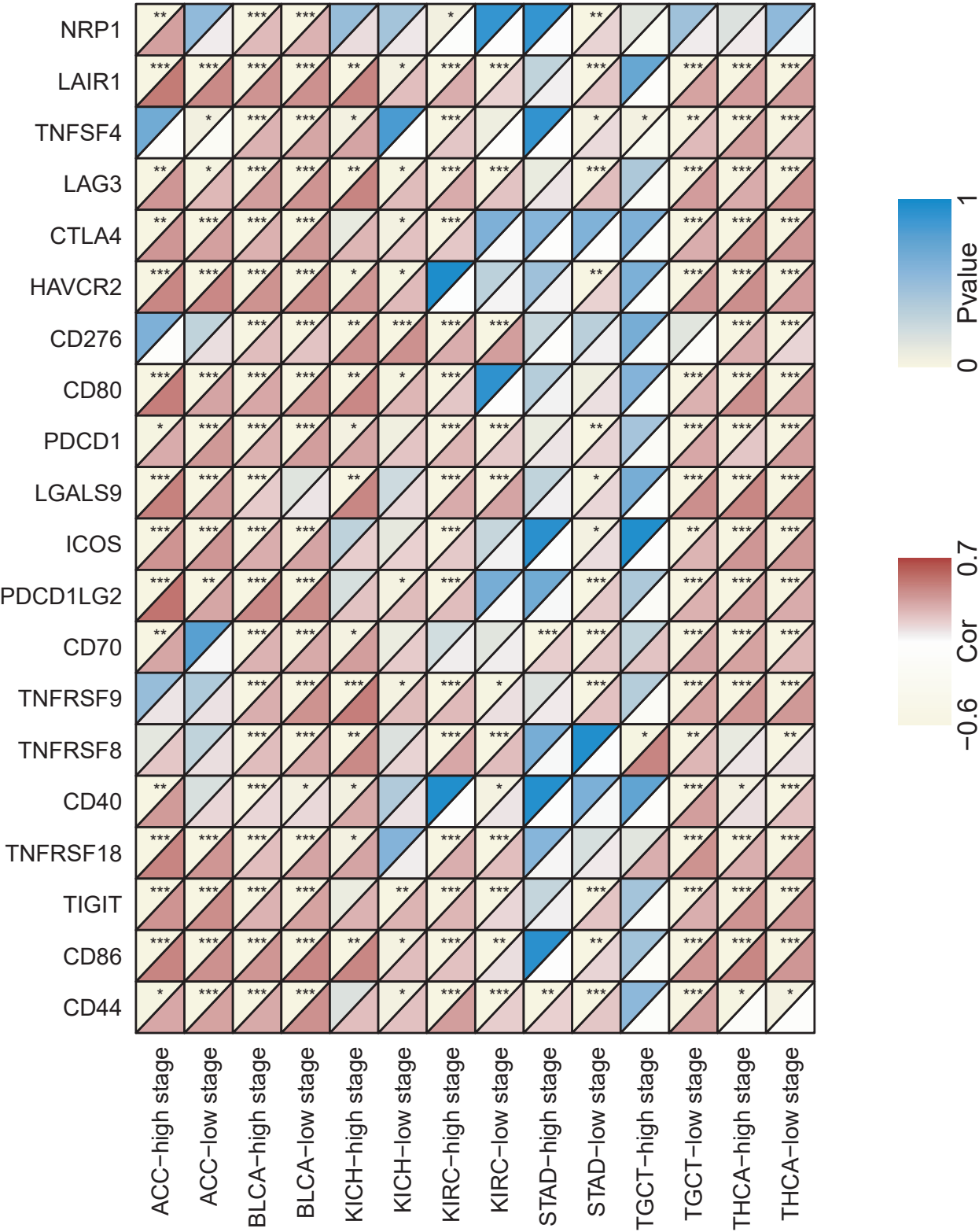

Supplement: Supplemental Material [file KBIE_A_1973220_SM3325.zip › supplementary/Figure S5.pdf]

**A**

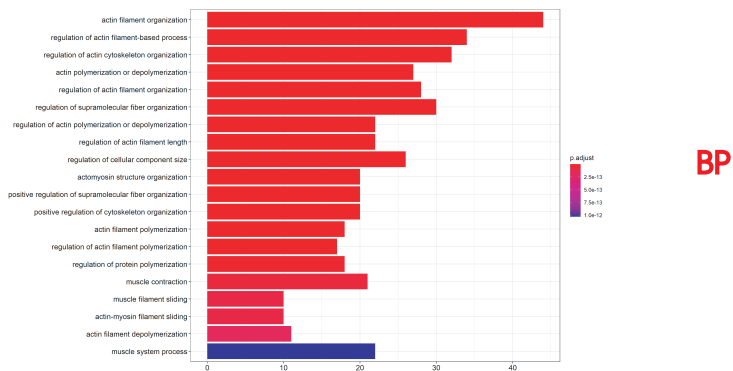

# B

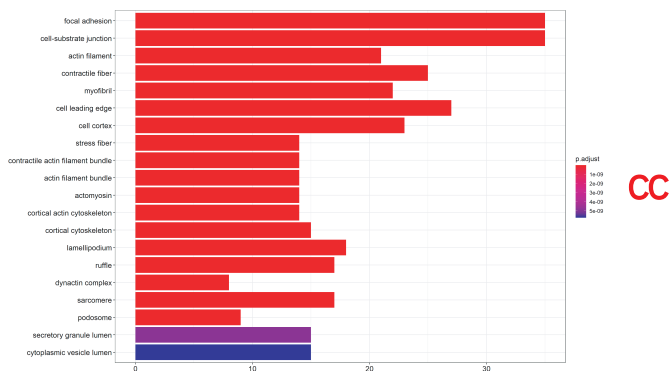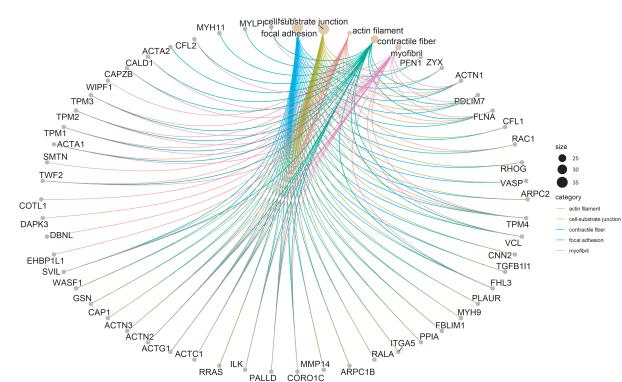

C

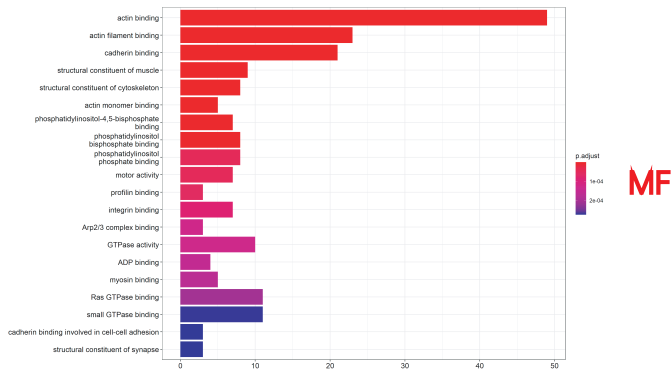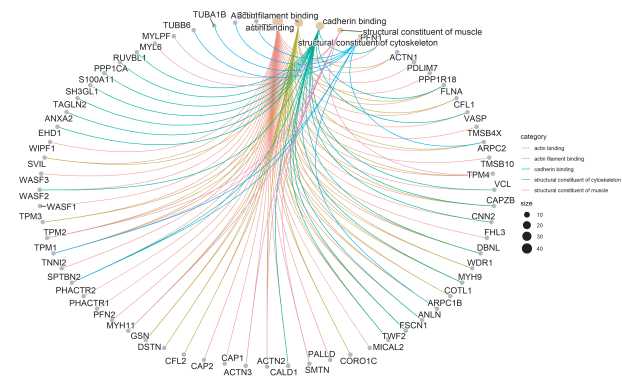

Supplement: Supplemental Material [file KBIE_A_1973220_SM3325.zip › supplementary/Figure S6.pdf]
